# Augmented reality navigation improves intraoperative resection accuracy but may not prevent alignment deviations in total knee arthroplasty

**DOI:** 10.1002/jeo2.70570

**Published:** 2025-12-18

**Authors:** Emmanuel Marchetti, Loïc Laurendon, ReSurg, Ahmed Assous, Chinyelum Agu, Jacobus H. Müller, Mo Saffarini, Antoine Combes, Roger Badet

**Affiliations:** ^1^ Centre OsteoArticulaire Fleming Bourgoin‐Jallieu France; ^2^ Medipole de Lyon Lyon France; ^3^ Clinique Saint Vincent de Paul Lyon France; ^4^ ReSurg SA Nyon Switzerland; ^5^ Clinique Saint Charles Lyon France

**Keywords:** AR, augmented reality, coronal alignment, TKA, total knee arthroplasty

## Abstract

**Purpose:**

The purpose was to compare intraoperative versus postoperative coronal, sagittal and axial alignment in total knee arthroplasty (TKA) performed using augmented reality (AR) navigation, and determine whether imperfect implant positioning affects postoperative alignment.

**Methods:**

A retrospective assessment was conducted on a study cohort of 70 patients (70 knees), who received unrestricted kinematic aligned TKA using AR navigation between February 2022 and April 2023. Implant positioning was assessed on postoperative frontal (divergence between the proximal tibial resection and baseplate) and sagittal radiographs (gaps between the distal femoral resection and implant) to distinguish between knees with adequate and imperfect implant positioning. The deviation between intraoperative and postoperative alignment measurements (lateral distal femoral angle (LDFA), medial proximal tibial angle (MPTA), hip knee ankle angle (HKA), posterior tibial slope (PTS) and femoral rotation) was assessed, and the number of outliers calculated using thresholds of 1° and 3°.

**Results:**

There were considerable proportions of knees with deviations between intraoperative and postoperative LDFA (>1°, 43 [61%]; >3°,14 [20%]), MPTA (>1°, 42 [60%]; >3°, 5 [7%]), HKA angle (>1°, 42 [60%]; >3°, 17 [24%]), and PTS (>1°, 47 [67%]; >3°, 15 [21%]). It is worth noting, however, that most of the relevant deviations were observed in knees that had imperfectly positioned implants (19 of 70, 27%), where the femoral component was inadequately impacted during surgery (15 of 19, 79%) and/or the tibial baseplate had an uneven cement distribution (8 of 19, 42%).

**Conclusion:**

The use of AR facilitates precise bone resections during TKA, yet it may not reduce deviations between intraoperative and postoperative LDFA, MPTA, HKA angle or PTS. As with any assistive technology, accurate bone resections alone are not sufficient to grant adequate implant positioning during TKA, which requires meticulous attention from surgeons to ensure sufficient component impaction and uniform cement distributions.

**Level of Evidence:**

IV.

**Abbreviations**
2D: two‐dimensional
AC: agreement coefficient
AR: augmented reality
CI: confidence interval
CT: computed tomography
FOV: field of view
HKA: hip‐knee‐ankle
ICC: intraclass correlation coefficient
LDFA: lateral distal femoral angle
MPTA: medial proximal tibial angle
PTS: posterior tibial slope

## INTRODUCTION

Numerous comparative studies on total knee arthroplasty (TKA) demonstrated considerably greater accuracy of bone resections when using assistive technologies such as navigation [3, 25, 26], patient‐specific instruments [20, 21], and robotics [1, 16, 17]. Despite accurate bone resections, implant and limb malalignment are sometimes observed following TKA, likely due to improper component insertion, inadequate impaction or uneven cement distribution [4]. Such imperfections in implant positioning could compromise knee function and contribute to patient dissatisfaction, observed in 7.5%–28.3% [12].

Augmented reality (AR) allows overlay of medical imaging onto the surgical field with simultaneous navigation via a head‐mounted display [13]. It is increasingly used in TKA, and has been shown to improve the accuracy of lateral distal femoral angle, medial proximal tibial angle and posterior tibial slope [5], though most studies were based on small samples [2, 11, 18, 19], focused on coronal joint alignment without considering overall limb alignment [4, 22, 23] or only used two‐dimensional (2D) imaging [4, 15, 19, 23, 24]. There are yet no published studies that investigated the accuracy of AR on axial plane alignment, nor the effects of imperfections in implant positioning on overall limb alignment.

The goals of the present study were therefore to (i) compare intraoperative versus postoperative coronal, sagittal and axial alignment in TKA performed using AR navigation, and (ii) determine whether imperfect implant positioning affects postoperative alignment. The hypotheses were that (i) joint and overall limb alignment would not be significantly different between intraoperative and postoperative coronal, sagittal and axial alignment, and (ii) imperfect implant positioning could affect postoperative alignment.

## MATERIALS AND METHODS

### Patients

The authors retrospectively reviewed the records of 83 patients (83 knees) who received unrestricted kinematic aligned TKA using AR between February 2022 and April 2023 by the senior surgeon (EM). All patients provided written informed consent to participate in the study and use their data and images for research purposes. The study was performed according to the guidelines of the Declaration of Helsinki and was approved by the institutional review board (IRB: COS‐RGDS‐2025‐05‐011‐MARCHETTI‐E).

Of the initial cohort of 83 patients, 12 had missing intraoperative data (not correctly backed up in the system), and one did not have postoperative scans. This left a study cohort of 70 patients (70 knees) (Table 1), of which 66 were operated for primary osteoarthritis and 4 were operated for post‐traumatic osteoarthritis.

**Table 1:** Characteristics of patients included in the analysis.

	Mean ± SD	
	*n* (%)	Range
Age	71.2 ± 6.8	53.0–84.0
BMI (kg/m^2^)	30.0 ± 4.6	19.0–42.0
Men	34 (49%)	
Women	36 (51%)	
Left	30 (43%)	
Right	40 (57%)	
Indication		
* Primary osteoarthritis*	66 (94%)	
* Secondary osteoarthritis* a	4 (6%)	

Abbreviations: BMI, body mass index; SD, standard deviation.

^a^ Post‐traumatic due to ACL rupture or fracture.

### Preoperative planning

Patients scheduled for TKA had preoperative computed tomography (CT) scans in a supine position with the leg in full extension and the foot secured to prevent motion. The field of view (FOV) captured the required bone regions (femoral head at the hip; femur, tibia, and fibula at the knee; and both malleoli at the ankle). The slice thickness was 1 mm with 0.7 mm overlap between slices. The acquired CT scans were exported as original DICOM files and then uploaded to measure lateral distal femoral angle (LDFA), medial proximal tibial angle (MPTA), hip knee ankle angle (HKA) and posterior tibial slope (PTS) femoral rotation.

### Surgical technique

The AR system (NextAR; Medacta, Switzerland) consisted of smart glasses, two small single‐use sensors, and a control unit. The smart glasses allowed for the display of the FOV real‐time information. The surgical navigation was performed by a wireless optical tracking system that included an active infrared camera and an active tracker that eliminated the need for external cameras. The tracker transmitted spatial data in six degrees of freedom (three translations, three rotations) with an error ≤0.5 mm/0.5°. The camera and tracker were attached to the femur and tibia within the surgical incision, eliminating the need for percutaneous bone pins [18].

A standard medial skin incision with a subvastus approach was performed. The system registered 26 points for each of the femur and tibia before removing any osteophytes, to align the CT scan with intraoperative bone surfaces. After registration, osteophytes were removed, and the reference lengths of the collateral ligaments were determined.

The cemented tibial component (GMK Primary 3, Medacta, Switzerland) was inserted, the final liner was positioned, and the uncemented femoral component (GMK Sphere, Medacta, Switzerland) was impacted. Final assessment was performed using the AR system, generating a detailed surgical report of bone cuts and ligament elongation [10, 18].

### Radiographic assessment

**Figure 1:**
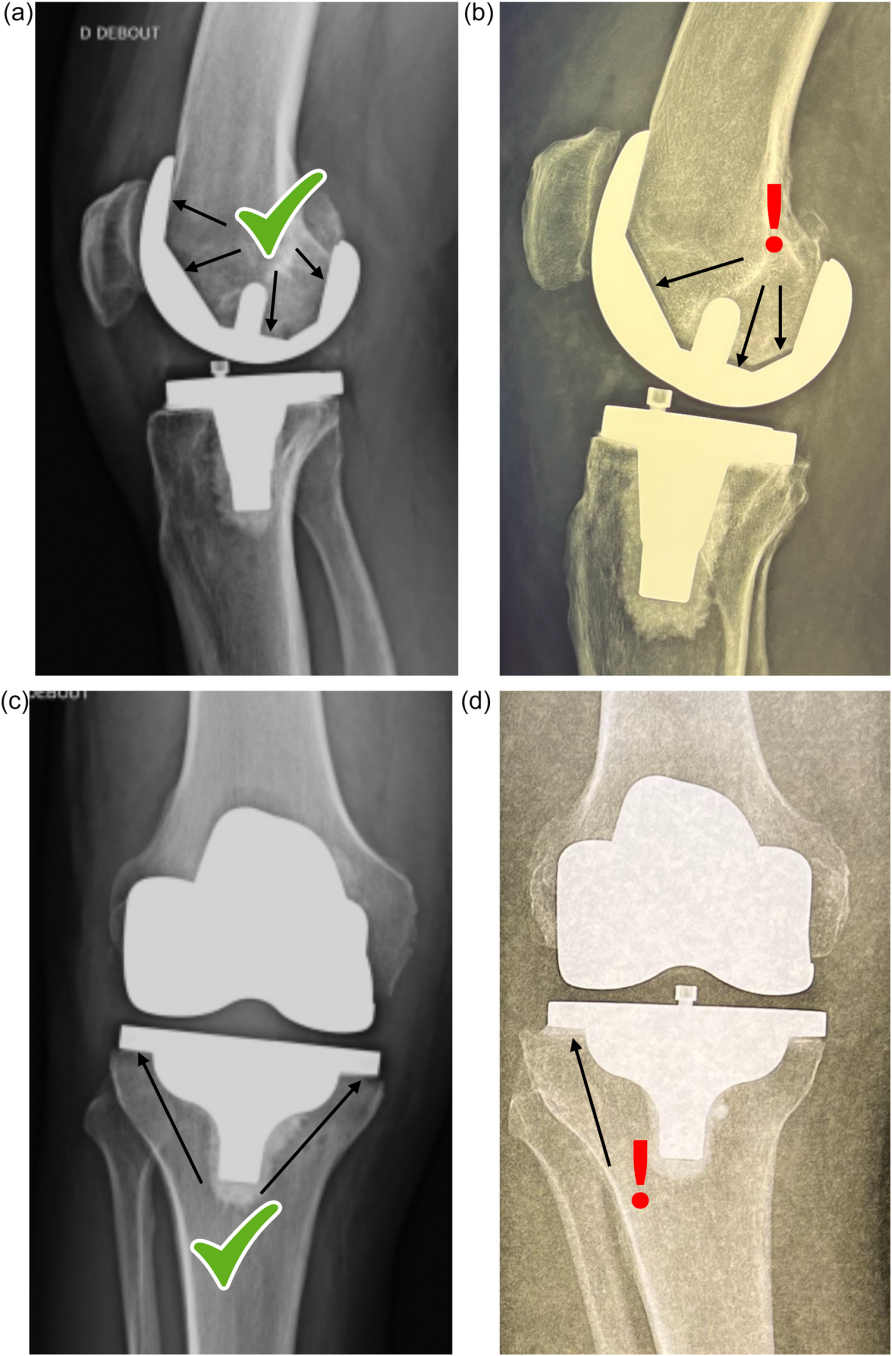
Radiographic assessment of adequate versus imperfect implant position. (a) Adequately positioned femoral component: No visible gaps between the distal femoral resection and femoral component on a sagittal view radiograph. (b) Imperfectly positioned femoral component: Visible gaps between the distal femoral resection and femoral component on a sagittal view radiograph. (c) Adequately positioned tibial baseplate: Uniform cement distribution between the proximal tibial resection and tibial baseplate component on a frontal view radiograph. (d) Imperfectly positioned tibial baseplate: Nonuniform cement distribution between the proximal tibial resection and tibial baseplate on a frontal view radiograph.

All patients received a postoperative CT scan, and a technician (Silvio Manca) measured LDFA, MPTA, HKA angle, PTS and femoral rotation. To assess the reliability of measurements, a second technician (Giuseppe Tranchida) independently measured the alignment of 49 knees. Intraclass correlation coefficients (ICC) with their 95% confidence intervals (95% CI) were calculated and were excellent for all angles, and therefore measurements from the first technician were used for the analysis (Table 2). Implant positioning was also assessed by the senior surgeon (EM) on postoperative frontal and sagittal radiographs to distinguish knees that had adequate implant positioning from those that had imperfect implant positioning (Figure 1). Imperfections in femoral implant positioning were mainly verified on sagittal radiographs, by inspecting for gaps between the distal femoral resection and metallic implant surface, likely due to inadequate component impaction during surgery. Imperfections in tibial implant positioning were mainly verified on frontal radiographs, by inspecting for divergence between the proximal tibial resection and the tibial baseplate surface, likely due to uneven cement distribution. To assess the reliability of this distinction, a second surgeon (LL) independently assessed all 70 knees. Gwet's agreement coefficient (AC) with its 95% CI was calculated and was excellent (Table 2).

**Table 2:** Inter‐observer reliability.

	ICC	95% CI
LDFA	0.95	0.91–0.97
MPTA	0.93	0.88–0.96
HKA angle	0.95	0.92–0.97
PTS	0.92	0.85–0.95
Femoral rotation	0.88	0.79–0.93
Implant positioning	0.96	0.84–1.00

Abbreviations: CI, confidence interval; HKA, hip knee ankle; ICC, intraclass correlation coefficient; LDFA, lateral femoral distal angle; MPTA, medial proximal tibial angle; PTS, posterior tibial slope.

### Data and statistical analysis

The normality of distributions was evaluated using the Shapiro‐Wilk test. Deviations between intraoperative and postoperative alignment measurements (LDFA, MPTA, HKA angle, PTS and femoral rotation) were assessed using the Student's *t*‐test (normal distributions) or Wilcoxon Rank Sum Test (skewed distributions). The authors also assessed the number and proportion of outliers using 1° and 3° thresholds for absolute deviations between intra‐ and post‐operative measurements. Differences in proportions of outliers between knees with adequately positioned and imperfectly positioned implants were assessed using the Chi‐squared test. All statistical analyses were performed using RStudio (2023.09.1 Build 494, Posit Software, PBC) and R (version 4.3.1, R Core Team). A value of *p* < 0.05 was considered statistically significant.

## RESULTS

In the study cohort of 70 knees, for LDFA, the absolute deviation was 1.7° ± 1.5° (*p* = 0.018); it exceeded 1° in 43 knees (61%), and exceeded 3° in 14 knees (20%). For MPTA, the absolute deviation was 1.4° ± 1.0° (*p* = 0.005); it exceeded 1° in 42 knees (60%), and exceeded 3° in 5 knees (7%). For the HKA angle, the absolute deviation was 2.2° ± 1.8° (*p* < 0.001); it exceeded 1° in 42 knees (60%), and exceeded 3° in 17 knees (24%). For PTS, the absolute deviation was 1.9° ± 1.6° (*p* < 0.001); it exceeded 1° in 47 knees (67%), and exceeded 3° in 15 knees (21%). For femoral rotation, the absolute deviation was 0.8° ± 0.7° (*p* = 0.008); it exceeded 1° in 18 knees (26%), and exceeded 3° in 2 knees (3%) (Table 3).

**Table 3:** Intra‐ and post‐operative joint and limb alignment of the entire cohort.

	Mean ± SD			
	*n*	%	Range	*p* value
LDFA				
Intraoperative	87.7 ± 1.7		−1.4 to 5.2	
Postoperative	88.6 ± 2.6		−4.0 to 8.0	
Mean deviation	0.9 ± 2.1		−3.7 to 6.0	0.018
Absolute deviation	1.7 ± 1.5		0.0–6.0	
>1° outliers	43	61%		
>3° outliers	14	20%		
MPTA				
Intraoperative	86.8 ± 1.8		−0.5 to 6.5	
Postoperative	85.8 ± 2.2		−0.5 to 8.5	
Mean deviation	−1.0 ± 1.5		−5.0 to 2.6	0.005
Absolute deviation	1.4 ± 1.0		0.0–5.0	
>1° outliers	42	60%		
>3° outliers	5	7%		
HKA angle				
Intraoperative	178.9 ± 1.9		175.0–183.0	
Postoperative	177.2 ± 2.8		171.0–183.5	
Mean deviation	1.7 ± 2.3		−4.5 to 7.0	<0.001
Absolute deviation	2.2 ± 1.8		0.0–7.0	
>1° outliers	42	60%		
>3° outliers	17	24%		
PTS				
Intraoperative	4.6 ± 1.7		−0.2 to 7.7	
Postoperative	5.9 ± 2.1		1.5–13.5	
Mean deviation	−1.3 ± 2.2		−10.3 to 3.1	<0.001
Absolute deviation	1.9 ± 1.6		0.1–10.3	
>1° outliers	47	67%		
>3° outliers	15	21%		
Femoral rotation^a^				
Intraoperative	0.2 ± 0.7		−1.1 to 2.5	
Postoperative	−0.1 ± 1.0		−2.0 to 4.0	
Mean deviation	0.3 ± 1.0		−2.8 to 3.2	0.008
Absolute deviation	0.8 ± 0.7		0.0–3.2	
>1° outliers	18	26%		
>3° outliers	2	3%		

Abbreviations: HKA, hip knee ankle; LDFA, lateral femoral distal angle; MPTA, medial proximal tibial angle; PTS, posterior tibial slope; SD, standard deviation.

^a^ Measured relative to native posterior condylar axis (PCA).

Of the study cohort of 70 knees, 51 (73%) were deemed to have adequately positioned implants, while 19 (27%) were deemed to have imperfectly positioned implants (11 femoral components, four tibial components and four both components). Comparing those two groups revealed that, for LDFA, absolute deviations were significantly greater for imperfectly (3.4° ± 1.7°) versus adequately (1.1° ± 0.8°) positioned implants (*p* < 0.001); deviations exceeding both 1° and 3° were also significantly greater for imperfectly positioned implants (*p* = 0.005 and *p* < 0.001). For MPTA, absolute deviations were similar for imperfectly (1.8° ± 1.5°) versus adequately (1.3° ± 0.7°) positioned implants (*p* = 0.297); only deviations exceeding 3° were significantly greater for imperfectly positioned implants (*p* = 0.001). For HKA angle, absolute deviations were significantly greater for imperfectly (4.2° ± 1.5°) versus adequately (1.4° ± 1.3°) positioned implants (*p* < 0.001); deviations exceeding both 1° and 3° were also significantly greater for imperfectly positioned implants (*p* < 0.001 and *p* < 0.001). For both PTS and femoral rotation, there were no significant differences in absolute deviations or number of outliers between adequately and imperfectly positioned implants (Table 4).

**Table 4:** Comparison of joint and limb alignment between adequately positioned and imperfectly positioned implants.

	Adequately positioned implants (*n* = 51)			Imperfectly positioned implants (*n* = 19)			
	Mean ± SD			Mean ± SD			
	*n*	%	Range	*n*	%	Range	*p* value
LDFA							
Intraoperative	88.0 ± 1.8		−1.4 to 5.2	87.2 ± 1.0		1.1–4.8	
Postoperative	88.3 ± 2.3		−3.0 to 6.0	89.7 ± 3.2		−4.0 to 8.0	
Mean deviation	0.3 ± 1.3		−2.1 to 2.9	2.5 ± 3.0		−3.7 to 6.0	<0.001
Absolute deviation	1.1 ± 0.8		0.0–2.9	3.4 ± 1.7		0.1–6.0	<0.001
>1° outliers	26	51%		17	89%		0.005
>3° outliers	0	0%		14	74%		<0.001
MPTA							
Intraoperative	87.0 ± 1.8		−0.5 to 6.5	86.1 ± 1.5		0.5–6.1	
Postoperative	86.2 ± 2.1		−0.5 to 8.5	84.8 ± 2.2		1.0–8.5	
Mean deviation	−0.8 ± 1.2		−2.9 to 2.6	−1.4 ± 1.9		−5.0 to 2.3	0.174
Absolute deviation	1.3 ± 0.7		0.0–2.9	1.8 ± 1.5		0.0–5.0	0.297
>1° outliers	30	59%		12	63%		0.790
>3° outliers	0	0%		5	26%		0.001
HKA angle							
Intraoperative	179.0 ± 2.1		175.0–183.0	178.9 ± 1.2		176.0–180.5	
Postoperative	178.0 ± 2.5		172.0–183.0	175.2 ± 2.9		171.0–183.5	
Mean deviation	1.0 ± 1.6		−4.5 to 4.0	3.7 ± 2.5		−3.0 to 7.0	<0.001
Absolute deviation	1.4 ± 1.3		0.0–4.5	4.2 ± 1.5		1.5–7.0	<0.001
>1° outliers	23	45%		19	100%		<0.001
>3° outliers	5	10%		12	63%		<0.001
PTS							
Intraoperative	4.6 ± 1.8		−0.2 to 7.7	4.5 ± 1.7		1.1–7.0	
Postoperative	5.9 ± 2.3		1.5–13.5	6.0 ± 1.7		2.5–8.5	
Mean deviation	−1.2 ± 2.4		−10.3 to 3.1	−1.5 ± 1.4		−3.6 to 1.0	0.405
Absolute deviation	2.0 ± 1.8		0.1–10.3	1.7 ± 1.1		0.4–3.6	0.787
>1° outliers	35	69%		12	63%		0.776
>3° outliers	11	22%		4	21%		1.000
Femoral rotation^a^							
Intraoperative	0.2 ± 0.7		−1.1 to 2.5	0.2 ± 0.8		−1.1 to 2.5	
Postoperative	0.0 ± 1.0		−1.5 to 4.0	−0.4 ± 0.7		−2.0 to 0.5	
Mean deviation	0.2 ± 0.9		−2.8 to 2.7	0.7 ± 1.1		−0.9 to 3.2	0.092
Absolute deviation	0.7 ± 0.6		0.0–2.8	1.0 ± 0.8		0.0–3.2	0.065
>1° outliers	11	22%		7	37%		0.440
>3° outliers	0	0%		2	11%		0.071

Abbreviations: HKA, hip knee ankle; LDFA, lateral femoral distal angle; MPTA, medial proximal tibial angle; PTS, posterior tibial slope; SD, standard deviation.

^a^ Measured relative to native posterior condylar axis (PCA).

## DISCUSSION

The most important findings of this study were that, even when using AR to improve accuracy of bone resections during TKA, considerable proportions of knees deviated by more than 3° between intraoperative and postoperative LDFA (20%), MPTA (7%), HKA angle (24%) and PTS (21%). It is worth noting, however, that most of the relevant deviations were observed in knees that had imperfectly positioned implants (19 of 70, 27%), where the femoral component was inadequately impacted during surgery (15 of 19, 79%) and/or the tibial baseplate had an uneven cement distribution (8 of 19, 42%). These findings refute the first hypothesis, as joint and overall limb alignment deviated significantly between intraoperative and postoperative coronal and sagittal alignment (but not axial alignment), but support the second hypothesis that imperfect implant positioning could affect postoperative alignment. The clinical relevance of these findings is that, as with any assistive technology, accurate bone resections alone are not sufficient to grant adequate implant positioning during TKA, which requires great attention from surgeons to ensure sufficient component impaction and uniform cement distributions.

The present study revealed substantial deviations in the sagittal and coronal planes; however, there were few, if any, in femoral axial rotation, probably due to the guidance provided by the box between the anterior and posterior femoral resections, which prevents rotational errors. Accurate control of femoral rotation is important to facilitate optimal flexion gap balancing as well as optimal patellar tracking [14]. To the authors’ knowledge, the present study was the first to assess the accuracy of femoral rotation using AR navigation. The other errors observed in the coronal and sagittal planes are difficult to avoid, especially when using a minimally invasive approach, where visibility and the working field are limited [27]. It would be beneficial, however, if implant manufacturers could integrate mechanisms built into the femoral and tibial components to optimise femoral impaction and tibial cement distribution (pegs, keels, fins, press‐fit tolerances).

A recent study by Lambrechts on 124 patients receiving AR‐assisted TKA with personalised alignment reported the number of postoperative outliers (>3°) from planned LDFA (8.5%), MPTA (2.5%), HKA angle (10.2%) and PTS (20.2%) [15]. The present study revealed that deviations by more than 3° decrease significantly when considering only adequately positioned implants; however, several studies report greater deviations in tibial slope compared to other coronal and axial alignments [15, 18, 19], which could highlight a technical challenge for achieving precise sagittal plane cuts using AR in TKA.

Two previous studies assessed radiographic outcomes in patients undergoing AR‐assisted TKA: the first evaluated only tibial coronal alignment (tibial varus) and PTS, and compared preoperative planned to intraoperative angles in 76 patients [4]. The mean difference between planned and intraoperative angles was 0.59° ± 0.55° for the tibial varus and 0.70° ± 0.75° for the PTS. The second study evaluated postoperative deviation of the LDFA from a neutral target and compared AR‐assisted TKA to accelerometer‐based TKA in 109 and 118 patients [24]. The absolute difference from neutral was 1.2° ± 1.0° in the AR‐assisted group (95.4% deviated <3°) and 1.3° ± 1.1° in the accelerometer‐based group (93.2° deviated <3°). As AR‐assisted TKA enables intraoperative assessment of a patient's coronal (LDFA, MPTA, HKA angle), sagittal (PTS) and axial alignment (femoral rotation), studies should ideally report all of these angles for objective evaluation, especially as it will allow tailoring TKA to a patient's phenotype [6, 7, 8, 9].

The findings of this present study should be interpreted with consideration of the following limitations. This study did not include a control group of surgery performed without AR or performed with an alternative assistive technology. Imperfect implant positioning was defined by visual inspection; although inter‐reader agreement was excellent based on the Gwet's AC, the assessment remains subjective. The distribution of knees with adequate versus imperfect implant positioning was unequal, and precluded regression analysis to explore potential confounders. Moreover, no a priori power analysis was performed because this was a retrospective study for which the number of eligible cases determined the sample size. The study was limited to one implant model and one surgeon, and therefore may not represent all TKA designs and surgeons in general. Finally, the authors did not assess the impact of deviations on functional outcomes, as the study included no patient‐reported outcomes or clinical scores.

## CONCLUSION

The use of AR facilitates precise bone resections during TKA, yet it may not reduce deviations between intraoperative and postoperative LDFA, MPTA, HKA angle, or PTS. As with any assistive technology, accurate bone resections alone are not sufficient to grant adequate implant positioning during TKA, which requires meticulous attention from surgeons to ensure sufficient component impaction and uniform cement distributions.

## CONTRIBUTORS OF ReSurg

Ahmed Assous, Chinyelum Agu, Jacobus H. Müller, and Mo Saffarini (ReSurg SA, Nyon, Switzerland).

## AUTHORS CONTRIBUTIONS

**Emmanuel Marchetti**: Conceptualisation; investigation; methodology; validation; writing—review and editing. **Loïc Laurendon**: Investigation; writing—review and editing. **ReSurg**: Statistical analysis; software; writing—original draft. **Antoine Combes**: Investigation; writing—review and editing. **Roger Badet**: Investigation; writing—review and editing.

## CONFLICT OF INTEREST STATEMENT

Emmanuel Marchetti reports personal fees from Medacta. Loïc Laurendon, Antoine Combes, Roger Badet, Ahmed Assous, Chinyelum Agu, Jacobus H. Müller, and Mo Saffarini have nothing to declare.

## ETHICS STATEMENT

The institutional review board approved the study in advance (IRB reference number: COS‐RGDS‐2025‐05‐011‐MARCHETTI‐E). All patients provided written informed consent to use their data and images for research and publishing purposes.

## Data Availability

Upon reasonable request, the authors can provide access to the data used for all analyses and analytic code.
